# Obstructive Sleep Apnea Following Head and Neck Chemoradiation: A Scoping Review With Exploratory Meta‐Analysis

**DOI:** 10.1002/hed.70163

**Published:** 2026-01-19

**Authors:** Augustin G. L. Vannier, Neil S. Kondamuri, Megan S. Wu, Janya Allen, Nihar Rama, Rachel Nordgren, Nishant Agrawal, Phillip S. LoSavio

**Affiliations:** ^1^ Pritzker School of Medicine University of Chicago Chicago Illinois USA; ^2^ Department of Surgery, Section of Otolaryngology—Head and Neck Surgery University of Chicago Chicago Illinois USA; ^3^ University of Chicago Chicago Illinois USA; ^4^ Department of Public Health Sciences University of Chicago Chicago Illinois USA

**Keywords:** cancer, chemotherapy, obstructive sleep apnea, radiotherapy

## Abstract

**Background:**

Obstructive sleep apnea (OSA) may contribute to fatigue in head and neck cancer patients undergoing chemotherapy and radiotherapy, particularly as both have the potential to impact the mechanics and dynamics of the airway. We conducted a scoping review and exploratory meta‐analysis to evaluate the risk of OSA following chemoradiation.

**Methods:**

PubMed, Web of Science, EMBASE, and Cochrane Library were searched for studies assessing incident OSA after radiotherapy or chemotherapy. Eligible studies isolated the effect of one treatment and reported OSA rates in each group.

**Results:**

Of 559 papers identified, 110 were duplicates and 30 underwent full‐text review; 13 met criteria for exploratory meta‐analysis. A modest, nonsignificant trend toward increased risk of mild OSA was observed with radiotherapy (*z* = 1.42, *p* = 0.15). Chemotherapy was not associated with mild (*z* = −0.83, *p* = 0.41) or moderate (*z* = 0.00, *p* = 1.00) OSA.

**Conclusions:**

Studies are limited in size and number, and do not support increased risk of OSA with radiotherapy.

## 1 Introduction

There is a significantly higher incidence of obstructive sleep apnea (OSA) among patients with head and neck cancer; however, the persistence of OSA after treatment with chemotherapy and radiotherapy remains poorly understood [1, 2, 3]. Head and neck cancer patients often complain of fatigue, particularly those patients who undergo head and neck chemotherapy and radiotherapy [4, 5, 6, 7, 8]. While fatigue may be related to the physical, emotional, and cognitive burden associated with cancer treatment, these treatment courses may also predispose patients to cancer‐related fatigue. Sleep disturbance, in particular, is common in patients with head and neck cancer, and may contribute to chronic fatigue [9], though it is a frequently overlooked comorbidity in head and neck cancer patients [10].

Pathophysiologically, space‐occupying lesions in head and neck cancers can cause mechanical, anatomic narrowing of the airway and disruption of the musculature that maintains airway patency, directly leading to obstructive sleep apnea. Indirectly, even distant cancers (e.g., esophageal) impose significant metabolic burden and cause non‐specific atrophy of skeletal muscle that may weaken airway musculature [11, 12]. Treatment may aggravate these effects. While surgical resection carries a direct risk if the cancer site neighbors the airway and surrounding tissues [3], chemotherapy and radiotherapy also induces direct tissue effect by causing metabolic disruption and inflammation, respectively [13]. These effects, in turn, may contribute to airway narrowing and weakening [13]. There are currently no systematic reviews evaluating the association between chemotherapy and the risk of developing obstructive sleep apnea [3, 14, 15], and numerous small‐scale reviews focused on radiotherapy provide conflicting evidence regarding the development of sleep apnea.

Given the conflicting evidence on the association of sleep apnea and nonsurgical cancer therapy, we performed a scoping review of the literature as well as an exploratory meta‐analysis of prospective and retrospective studies evaluating the risk of developing obstructive sleep apnea in patients who receive chemotherapy or radiotherapy for head and neck cancer. As long‐term morbidity of sleep apnea includes cardiovascular comorbidities, stroke, and even early mortality [16, 17], and treatment options for sleep apnea have advanced to include neurostimulation procedures performed by otolaryngologists with excellent outcomes, defining the relationship between cancer‐related fatigue and sleep apnea is important for providing optimal care for head and neck cancer patients.

## 2 Materials and Methods

### 2.1 Search Strategy

**FIGURE 1:**
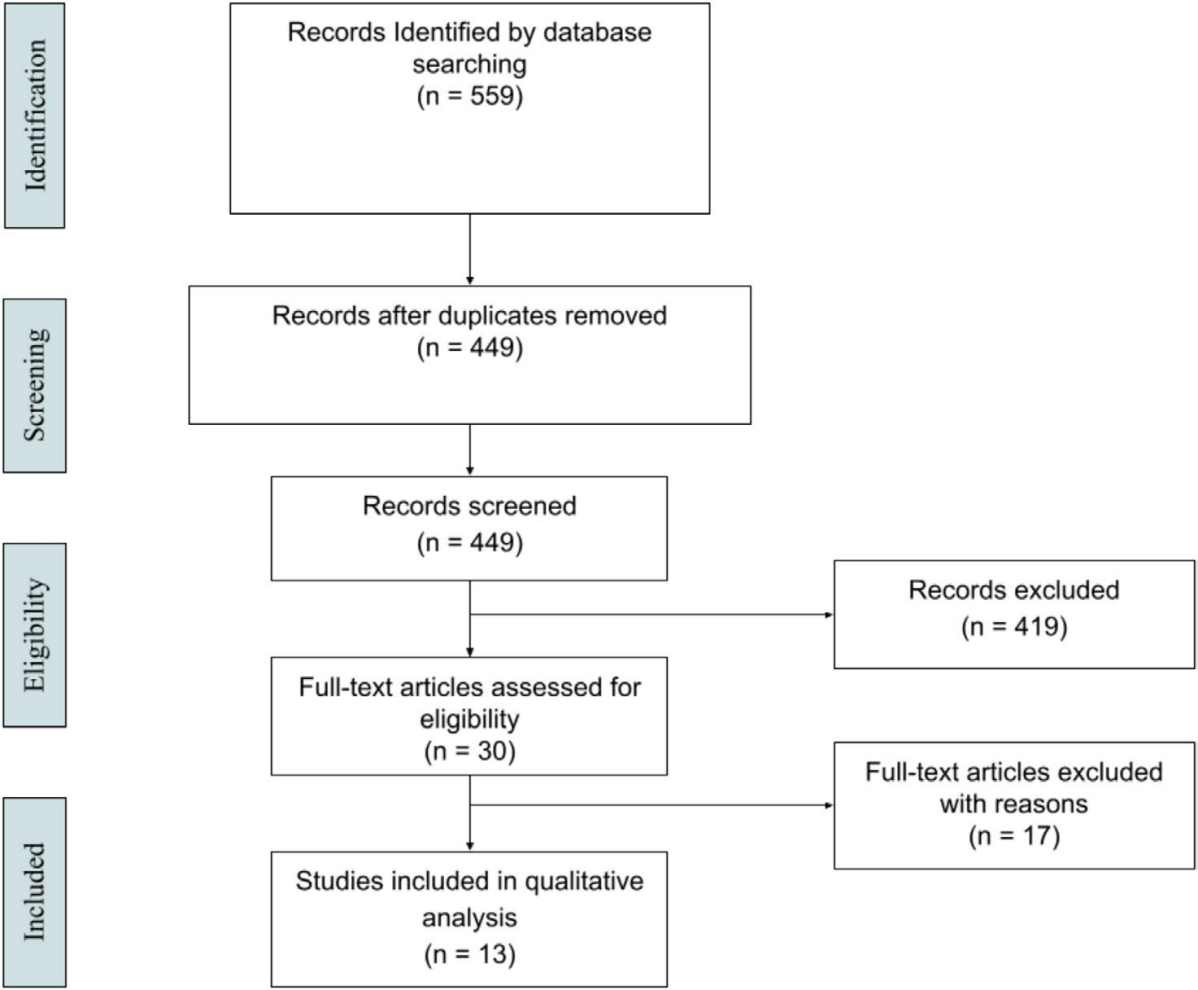
PRISMA flow diagram. PRISMA flow diagram showing studies included and excluded at each stage of review. *n*: number. [Color figure can be viewed at wileyonlinelibrary.com]

We conducted a systematic search of PubMed, Web of Science, EMBASE and the Cochrane Library using the following Medical Subject Headings (MeSH) terms: “cancer, cancerous, neoplasm”; “obstructive sleep apnea, sleep apnea, apnea”; “chemotherapy, radiotherapy.” Different combinations of these key terms were used to identify relevant studies related to the risk of OSA following radiotherapy or chemotherapy for any cancer. A complete list of search terms is included in the Table S1. The search strategy was executed on 8/3/2024. The protocol was based on Preferred Reporting Items for Systematic Reviews and Meta‐Analysis (PRISMA) guidelines (Figure 1). This study received exempt determination prior to analysis from the University of Chicago Institutional Review Board.

Papers were included if therapy type was described and either radiotherapy or chemotherapy was included as a treatment option. Randomized controlled trials, prospective cohort studies, and retrospective cohort studies were included. Duplicate articles, conference abstracts, book chapters, books, case reports, editorial letters, review articles, single‐arm studies, and opinion papers were excluded. Additionally, studies in other languages, in pediatric populations, and without measures of statistical significance [hazard ratios (HR), relative risk (RR), or odds ratio (OR) with confidence intervals (CI)] were excluded. Lastly, papers focused on non‐head and neck cancers and those without data regarding OSA outcomes following treatment were excluded.

### 2.2 Data Extraction

A two‐pass screen was conducted with two authors (A.V., N.K.) assessing titles and abstracts for inclusion. Any disagreements were resolved by the senior author (P.S.L.). Full‐text articles were then independently reviewed by the two authors (A.V., N.K.) with any disagreements resolved by the senior author (P.S.L.). Common reasons for exclusion were non‐relevant study subject, excluded study type (e.g., conference abstract, review), and a lack of analyzable data published in the study. Two authors (A.V., N.K.) independently extracted the following data from the following PRISMA 2020 guidelines: article information (authors, year of publication, journal, study title), study information (study design, sample size, radiotherapy vs. chemotherapy exposure), patient demographics (gender and age), and outcomes of mean polysomnogram values of apnea‐hypopnea index (AHI). Information regarding other sleep apnea variables including lowest oxygen saturation (LSAT), oxygen desaturation index (ODI), and Epworth Sleepiness Scale (ESS) scores was extracted.

### 2.3 Statistical Analyses

Descriptive statistics were performed using python. Meta‐analytics were performed with the IBM Statistical Package for Social Sciences software version 29.0 (IBM, Armonk, NY) as well as with the Review Manager (RevMan) version 7.2.0 software (The Cochrane Collaboration, London, United Kingdom). RevMan was used to calculate the relative risk (RR), mean difference (MD), and 95% confidence interval (95% CI) of both metrics. For both paired and unpaired two‐tailed *t* tests, a *p* value < 0.05 was considered statistically significant. An exploratory meta‐analysis was performed using random effects modeling. A funnel plot assessment was performed with Egger's regression wherever five studies or greater could be aggregated; multiplicative residual heterogeneity variance (τ^2^) as well as bias (estimate, standard error, and *p*‐value) are reported [18].

## 3 Results

**FIGURE 2:**
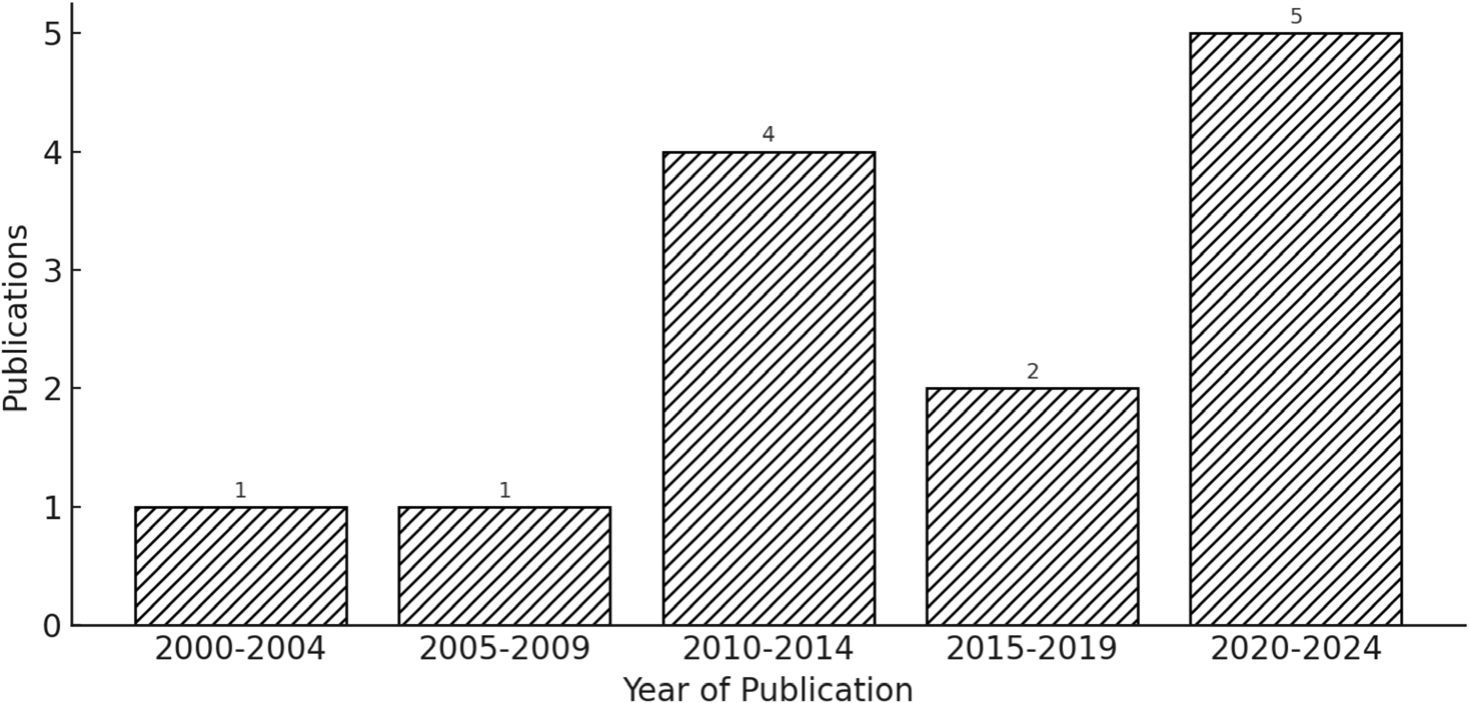
Publishing trend over time. Bar diagram showing number of publications per year meeting inclusion criteria from 2000 to 2024.

The initial review yielded 559 articles in the PubMed, Web of Science, Embase, and Cochrane Review databases (Table S1). One hundred and ten articles were duplicates, and 449 were screened for eligibility. Thirty papers met criteria for full text review, of which 13 were considered eligible for analysis (Figure 1). Of these, all 13 had treatment groups that were comparable and included in analysis (Table 1). Three non‐head and neck studies were discovered and excluded. The plurality of single‐site studies examined head and neck cancers in the oropharynx (*n* = 2; 15.4%) and larynx (*n* = 2; 15.4%) (Table 2). Only one study was dedicated to evaluation of treatment of oral cavity cancers alone. The 13 studies eligible for analysis were published between 2001–2024, with over half (*n* = 7; 53.8%) appearing in 2017 or later (Figure 2). Fewer than half had prospective cohort design (*n* = 6; 46.2%). The search revealed no randomized controlled trials studying OSA as an outcome of the application of radiotherapy or chemotherapy. Most studies were small: the median [IQR] sample size was 26 [21–56]. Existing studies used heterogeneous definitions of sleep apnea, including AHI (*n* = 8), ODI (*n* = 1), RDI (*n* = 2), diagnosis of OSA (*n* = 1), suspected OSA (*n* = 1). Where reporting of OSA diagnosis was made using binary AHI cutoffs, standard AHI definitions of mild, moderate, and severe OSA were not uniformly utilized [19, 20]. Findings from exploratory meta‐analyses are detailed below.

**TABLE 1:** Publications meeting inclusion criteria.

Year	1st author	Study type	OSA definition	Total patients	Mean age (SD)	Median age (IQR)	Gender (% male)	Patients with OSA (% of total)
2001	Friedman	Prospective	RDI > 15	24	64.8 (−)	—	87.5	21 (87.5)
2009	Steffen	Prospective	AHI > 20	31	64.48 (−)	—	71.0	6 (19.4)
2010	Qian	Retrospective	Raw RDI, RDI > 15 here	24	60.7 (−)	—	66.7	13 (54.2)
2012	Chan	Retrospective	AHI > 5	26	52.0 (9.9)	—	92.3	14 (53.8)
2014	Faiz	Retrospective	AHI > 5	56		60 (−)	76.7	47 (83.9)
2014	Lin	Retrospective	Raw AHI	18	49.8 (−)	—	77.8	—
2017	Huyett	Prospective	AHI > 5	16	62.1 (9.7)	62 (18.5)	81.2	14 (87.5)
2017	Loth	Prospective	AHI > 10	61	61.1 (−)	—	60.7	16 (26.2)
2021	Saesen^a^	Retrospective	Suspected OSA	50	64.2 (−)	—	66.0	20 (40.0)
2021	Nguyen	Prospective	AHI > 15, Raw AHI, Raw ESS	20	63.45 (7.5)	64.5 (12.5)	75.0	11 (55.0%)
2022	Inoshita	Prospective	AHI > 5, AHI > 30, PSG elements, ESS	21	62.2 (12.7)	—	95.2	—
2023	Chen	Retrospective	AHI > 5	59	60.1 (8.53)	—	93.2	44 (74.6)
2024	Karsten	Retrospective	OSA diagnosis	67	66 (−)	66 (−)	65.7	48 (71.6)

Abbreviations: AHI, apnea‐hypoapnea index; ESS, Epworth sleepiness scale; HN, head and neck cancer; IQR, interquartile range; OSA, obstructive sleep apnea; PSG, polysomnogram; RDI, respiratory disturbance index; SD, standard deviation.

^a^ Paper not included in meta‐analysis because definition of OSA was not comparable across papers (OSA suspected based on questionnaire response).

**TABLE 2:** Sites of head and neck cancers included in each study.

Head and neck cancer site	Author	Year
Nasopharynx (7.7%)	Lin	2014
Oral cavity (7.7%)	Chan	2012
Oropharynx (15.4%)	Qian	2010
	Loth	2017
Larynx (15.4%)	Nguyen	2021
	Chen	2023
Mixed^b^ (46.2%)	Friedman	2001
	Steffen	2009
	Faiz	2014
	Huyett	2017
	Saesen^a^	2021
	Inoshita	2022
	Karsten	2024

^a^ Papers not included in meta‐analysis.

^b^ Mixed papers examined nasopharynx, oral cavity, oropharynx, and laryngeal sites of head and neck cancer.

### 3.1 Risk of Developing OSA Following Radiotherapy

**FIGURE 3:**
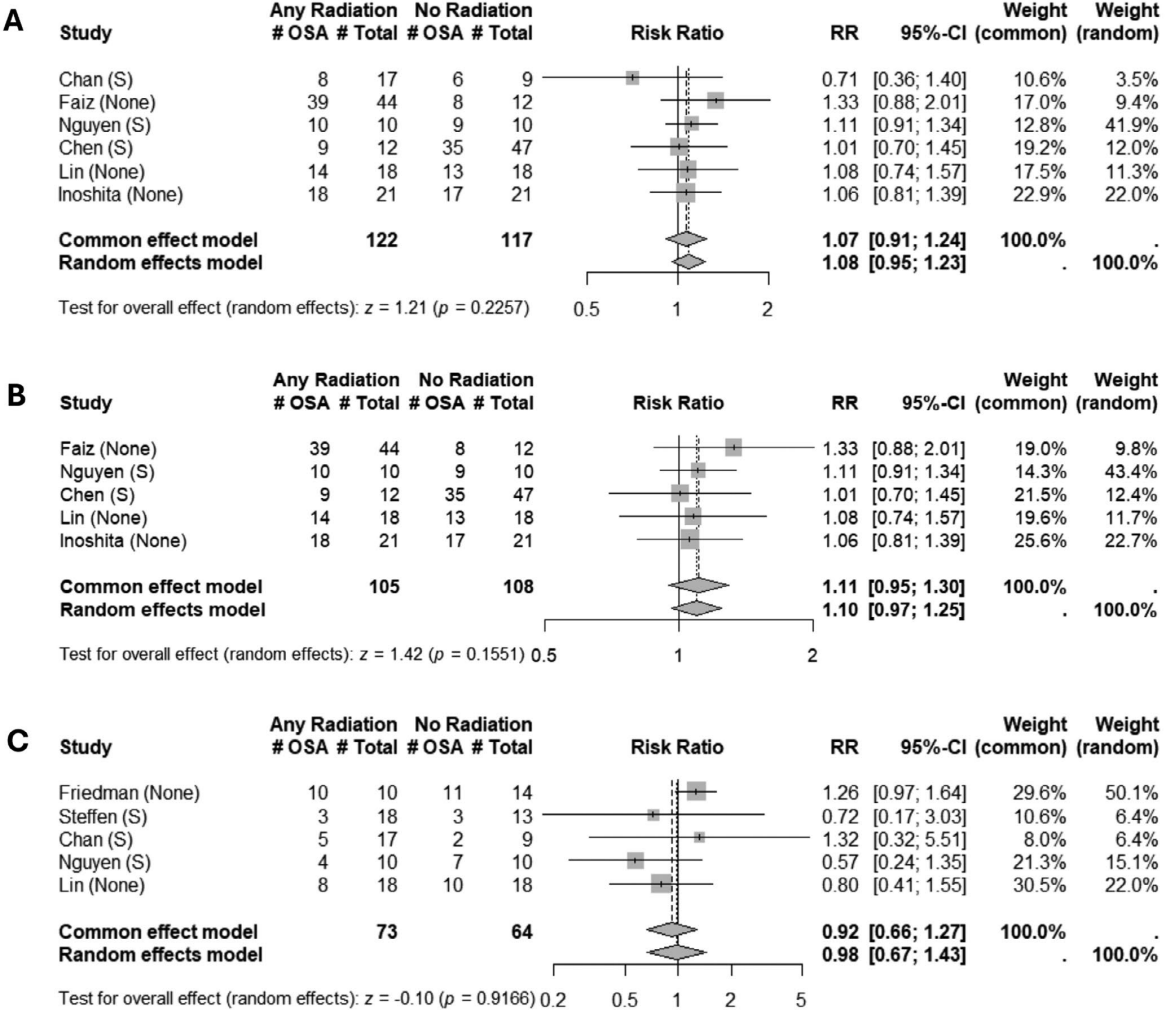
Association of radiation with development of obstructive sleep apnea. Forest plot for the risk of (A) mild OSA, (B) mild OSA excluding oral cavity cancers, and (C) moderate OSA following radiotherapy. Each study indicates the name of the study included, and the comparator treatment group against which the effect of treatment on OSA is isolated. AHI, apnea‐hypopnea index; CI, confidence interval; OSA, obstructive sleep apnea; S, surgery.

Eight studies examined the risk of developing OSA following radiation therapy. For the risk of developing mild OSA, six studies representing 239 patients were included. Three of six studies isolated the effect of radiotherapy after patients received surgical management of their disease, while the other three examined the effect of radiotherapy versus no treatment. While there was a trend toward development of mild OSA noted, this was not significant (RR: 1.08 [0.95; 1.23], *z* = 1.21, *p* = 0.23) (Figure 3A). One notable outlier, a paper by Chan et al., demonstrated decreased likelihood of OSA after radiation [21]. In excluding this outlier, the risk of developing mild OSA trended toward significance, though remained non‐significant (RR: 1.10 [0.97; 1.25], *z* = 1.42, *p* = 0.15) (Figure 3B). In the risk of developing moderate OSA, five studies representing 137 patients were included. Three of five studies isolated the effect of radiotherapy against a background of surgery, while the other two isolated the effect of radiotherapy versus no treatment. There was no association between receiving radiotherapy and the risk of developing moderate OSA (RR: 0.98 [0.67; 1.43], *z* = −0.10, *p* = 0.92) (Figure 3C). Among sleep study metrics, four studies reported AHI, three reported LSAT, two reported ODI, and two reported ESS.

### 3.2 Risk of Developing OSA Following Chemotherapy

**FIGURE 4:**
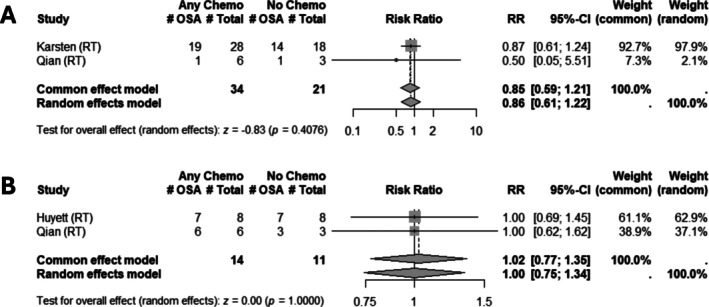
Association of chemotherapy with development of obstructive sleep apnea. Forest plot for the risk of (A) mild and (B) moderate obstructive sleep apnea following chemotherapy. Each study indicates the name of the study included, and the comparator treatment group against which the effect of treatment on OSA is isolated. AHI, apnea‐hypopnea index; CI, confidence interval; OSA, obstructive sleep apnea; RT, radiotherapy; SD, standard deviation.

Three studies examined the risk of developing OSA following chemotherapy. For the risk of developing mild OSA, two studies representing a combined 25 patients were included. Both studies isolated the effect of chemotherapy in the setting of patients receiving chemoradiation. No trend was identified for the impact of chemotherapy on the risk of developing mild OSA (RR: 1.00 [0.75; 1.34], *z* = −0.00, *p* = 1.00) (Figure 4A). In examining the risk of developing moderate OSA, two studies representing a combined 55 patients were included, both isolating the effect of chemotherapy in the setting of patients receiving chemoradiation. There was no association between receiving chemotherapy and the risk of developing moderate OSA (RR: 0.86 [0.61; 1.22], *z* = −0.83, *p* = 0.41) (Figure 4B). Among sleep study metrics, three studies reported AHI, and two reported LSAT. ESS and ODI were reported in only one study each. All funnel plots are reported in Figure S1.

## 4 Discussion

In this study, we systematically reviewed and synthesized the existing literature on the risk of developing obstructive sleep apnea in patients undergoing head and neck chemoradiation. These findings highlight increased interest in OSA outcomes over time and a modest, nonsignificant trend toward increased risk of mild OSA among patients receiving radiotherapy, though numerous limitations in existing data preclude the ability to assess true changes in sleep‐disordered breathing. Overall, we find that the current literature has small sample sizes that are underpowered to detect significant differences in OSA incidence with poorly standardized reporting of outcomes. Taken together, these results suggest larger studies are needed to determine whether chemoradiation impacts post‐treatment obstructive sleep apnea for survivors of head and neck cancer.

Findings of the exploratory meta‐analysis fit within the broader context of research exploring sleep apnea in head and neck care. Three systematic reviews and meta‐analyses have examined the association of OSA with radiotherapy for head and neck cancer with three differing conclusions [3, 14, 15]. One finds that radiation may influence the risk of developing OSA, another finds that only a minority of studies find an effect of treatment modality, and the third finds a notable, though nonsignificant, trend toward increased rates of sleep apnea in patients treated with radiotherapy [3, 14]. The present study is the first review to assess the impact of chemotherapy on risk of developing OSA, and we do not find a significant association between receiving chemotherapy and the risk of developing OSA.

Obstructive sleep apnea is a growing area of interest in head and neck cancer survivorship, consistent with the growing frequency of publications on the subject found by this study. A recent American Head and Neck Society consensus statement highlighted the effect of fatigue on the quality of life of patients undergoing chemoradiation [22]. Concerns are also emerging that intermittent hypoxia secondary to obstructive sleep apnea may directly contribute to treatment resistance or failure [23]. Despite growing interest, nonstandard methodologies and definitions of sleep apnea continue to hamper this area of investigation. For rigorous analysis, sleep studies before and after cancer treatment will be necessary to assess post‐treatment sleep apnea burden.

The divergence of study findings may have a biological explanation, as chemoradiation has a heterogeneous effect on the risk of developing OSA; the interplay between tumor burden, treatment‐related changes, and airway dynamics is complex [24, 25]. The reduction of tumor bulk in or adjacent to the airway through surgery, radiotherapy, or chemotherapy can mechanically relieve obstruction, potentially improving airway patency as well as pre‐existing OSA in some patients [24]. However, this benefit may be counterbalanced by treatment‐induced effects that contribute to airway dysfunction. Radiotherapy, for instance, can lead to fibrosis, scarring, and tissue stiffness, which may reduce the flexibility of airway structures and impair neuromuscular control [25]. Indeed, in this study, we note that while radiotherapy for tongue cancers seemed to improve AHI in Chan et al. which was the only study to exclusively examine cancer of the tongue, all other studies demonstrated worsening sleep apnea following radiotherapy. It is therefore plausible that the effect of radiotherapy may be site‐specific. Meanwhile, chemotherapy can induce systemic muscle wasting, including atrophy of pharyngeal musculature, which may predispose to airway collapse. Both chemotherapy and radiation further contribute to chronic inflammation, which can exacerbate soft tissue edema and compromise upper airway stability. Given these competing factors, the net impact of cancer therapy on OSA risk likely varies between individuals and depends on tumor location, baseline airway anatomy, and specific treatment regimen. This complexity underscores the need for larger, longitudinal studies that assess both short‐ and long‐term changes in airway function among cancer survivors. As sleep apnea is associated with development of cardiometabolic disease and early mortality [16, 26, 27], clarifying treatment effect may be critical in improving quality of life and survivorship in head and neck cancer patients.

This study has several limitations. Chiefly, current literature is limited to small‐scale studies with heterogeneous treatment groups, complicating the isolation of a given treatment in a like‐for‐like comparison [1, 2, 19, 20, 21, 28, 29, 30, 31, 32, 33, 34, 35, 36, 37, 38]. There are currently no large‐scale studies, and existing studies are underpowered to detect even moderate effect sizes, even when multiple studies are aggregated. Thus, the existing literature of small studies mandates further research on the effect of cancer therapy on OSA. We are not able to extract consistent data from analyzed studies, such as body mass index, to assess possible variation in comorbidities between studies. Moreover, existing studies vary in important ways, making it difficult to come to meaningful conclusions: tumor site, adjuvant treatment modalities, timing of post‐treatment sleep study, type of sleep study performed all vary between studies. Exploratory meta‐analysis is further limited by heterogeneous reporting of OSA based on apnea‐hypopnea index (with different cutoffs defining sleep apnea), respiratory disturbance index, Epworth sleepiness scale, suspected sleep apnea, and observed apneic events. Where sleep studies are performed, most odds ratios compare patients who receive chemotherapy or radiation to patients who do not, and therefore cannot reflect pre‐ to post‐treatment changes. Lastly, we limited the scope to studies of sleep disordered breathing, and were therefore unable to evaluate the impact of cancer therapies on other causes of treatment‐associated fatigue, such as insomnia or parasomnias. In the absence of an observed OSA‐mediated contribution to treatment‐associated fatigue, exploration of alternative causes is warranted.

## 5 Conclusion

Our scoping review finds existing literature is limited to small‐scale studies underpowered to detect moderate effect sizes. Exploratory meta‐analysis finds a small, nonsignificant effect of radiation on risk of developing mild sleep apnea, consistent with other literature, though finds no effect of chemotherapy on the risk of developing OSA. Site‐specific differences in the head and neck demonstrate that large‐scale prospective studies are warranted, with subgroup analyses to differentiate cancers based on primary tumor site.

## Funding

The authors have nothing to report.

## Disclosure

As a scoping review of existing studies, this work does not use human subjects and is IRB exempt at the University of Chicago.

## Conflicts of Interest

The authors declare no conflicts of interest.

## Supporting information


**Table S1:** MeSH search terms.
**Figure S1:** Funnel plots and heterogeneity estimates of studies evaluating the risk of OSA following radiotherapy.

## Data Availability

The data that support the findings of this study are available from the corresponding author upon reasonable request.
