# Supplementary material for: Obstructive Sleep Apnea Following Head and Neck Chemoradiation: A Scoping Review With Exploratory Meta‐Analysis
Source: Head Neck. 2026 Jan 19;48(6):1587–94. doi: 10.1002/hed.70163 (PMC13155189; doi:10.1002/hed.70163)

**SUPPLEMENTARY MATERIAL**

**Supplementary Table 1**: MeSH search terms

| **Database** | **Cancer Terms (OR)** | **AND** | **Obstructive Sleep Apnea Terms (OR)** | **AND** | **Therapy Terms (OR)** |
| --- | --- | --- | --- | --- | --- |
| **PubMed (85 titles)** | "cancer s"[All Fields] OR "cancerous"[All Fields] OR "neoplasms"[MeSH Terms] OR "neoplasms"[All Fields] OR "cancer"[All Fields] OR "cancers"[All Fields] | AND | "obstructive sleep apnoea"[All Fields] OR "sleep apnea, obstructive"[MeSH Terms] OR ("sleep"[All Fields] AND "apnea"[All Fields] AND "obstructive"[All Fields]) OR "obstructive sleep apnea"[All Fields] OR ("obstructive"[All Fields] AND "sleep"[All Fields] AND "apnea"[All Fields]) | AND | "chemotherapy s"[All Fields] OR "chemotherapies"[All Fields] OR "chemotherapy"[All Fields] OR "radiotherapy"[MeSH Terms] OR "radiotherapy"[All Fields] OR "radiotherapies"[All Fields] OR "radiotherapy"[MeSH Subheading] OR "radiotherapy s"[All Fields] OR ("radiation"[All Fields] AND "therapy"[All Fields]) OR "radiation therapy"[All Fields] OR "radiation therapies"[All Fields] |
| **Web of Science (94 titles)** | ALL=("cancer s"[All Fields] OR "cancerous"[All Fields] OR "neoplasms"[MeSH Terms] OR "neoplasms"[All Fields] OR "cancer"[All Fields] OR "cancers"[All Fields]) | AND | "obstructive sleep apnoea"[All Fields] OR "sleep apnea, obstructive"[MeSH Terms] OR ("sleep"[All Fields] AND "apnea"[All Fields] AND "obstructive"[All Fields]) OR "obstructive sleep apnea"[All Fields] OR ("obstructive"[All Fields] AND "sleep"[All Fields] AND "apnea"[All Fields]) | AND | ("chemotherapy s"[All Fields] OR "chemotherapies"[All Fields] OR "chemotherapy"[All Fields]) OR ("radiotherapy"[MeSH Terms] OR "radiotherapy"[All Fields] OR "radiotherapies"[All Fields] OR "radiotherapy"[MeSH Subheading] OR "radiotherapy s"[All Fields] OR ("radiation"[All Fields] AND "therapy"[All Fields]) OR "radiation therapy"[All Fields] OR "radiation therapies"[All Fields]) |
| **Embase (408 titles)** | 'cancer s' OR 'cancerous' OR 'neoplasm'/exp OR 'neoplasms' OR 'cancer' OR 'cancers' | AND | ('obstructive sleep apnoea' OR 'obstructive sleep apnea'/exp OR ('sleep' AND 'apnea' AND 'obstructive') OR 'obstructive sleep apnea' OR ('obstructive' AND 'sleep' AND 'apnea')) | AND | 'chemotherapy s' OR 'chemotherapies' OR 'chemotherapy' OR 'radiotherapy'/exp OR 'radiotherapies' OR 'radiotherapy' OR 'radiotherapy s' OR ('radiation' AND 'therapy') OR 'radiation therapy' OR 'radiation therapies' |
| **Cochrane (16 titles)** | cancer | AND | sleep apnea |  | chemotherapy OR radiation therapy OR radiotherapy |

**Supplementary Figure 1**: Funnel plots and heterogeneity estimates of studies evaluating the risk of OSA following radiotherapy

*Funnel plots for studies evaluating the effect of radiotherapy on OSA for mild OSA (A), mild OSA without Chan et. Al and moderate OSA (C) Abbreviations: obstructive sleep apnea (OSA)*

A B


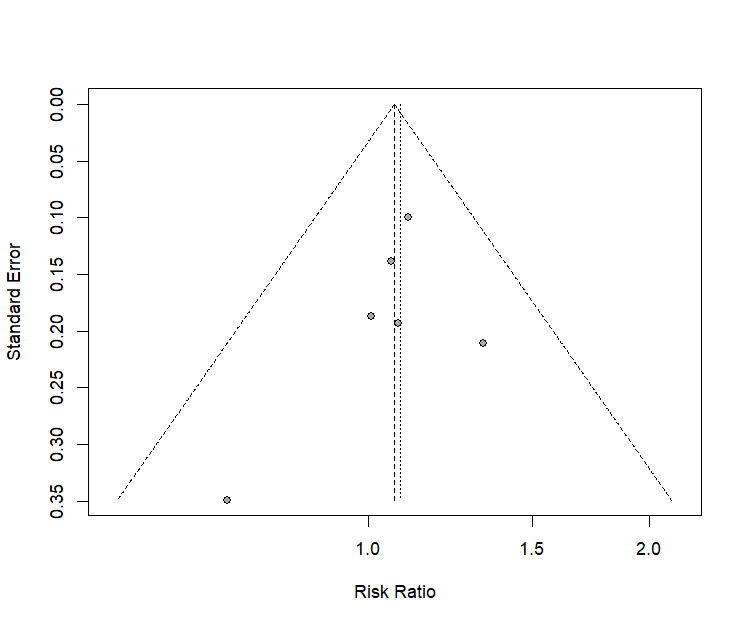

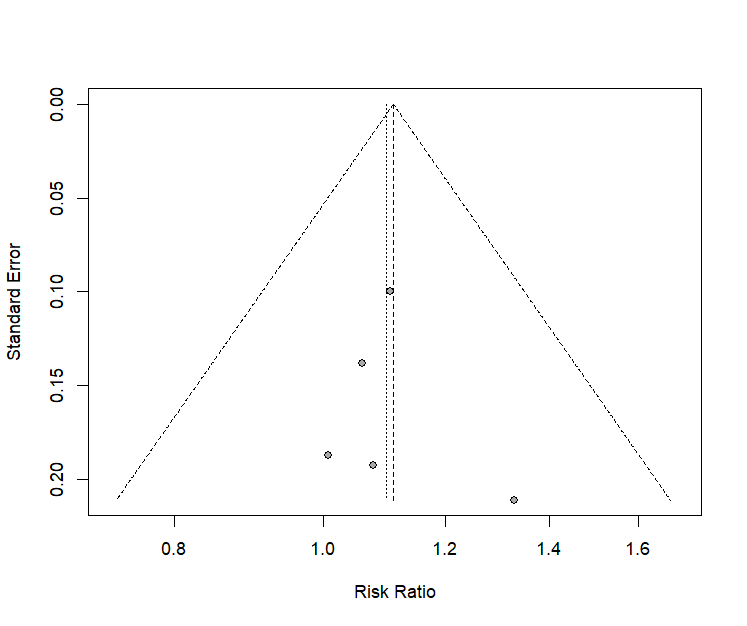


C


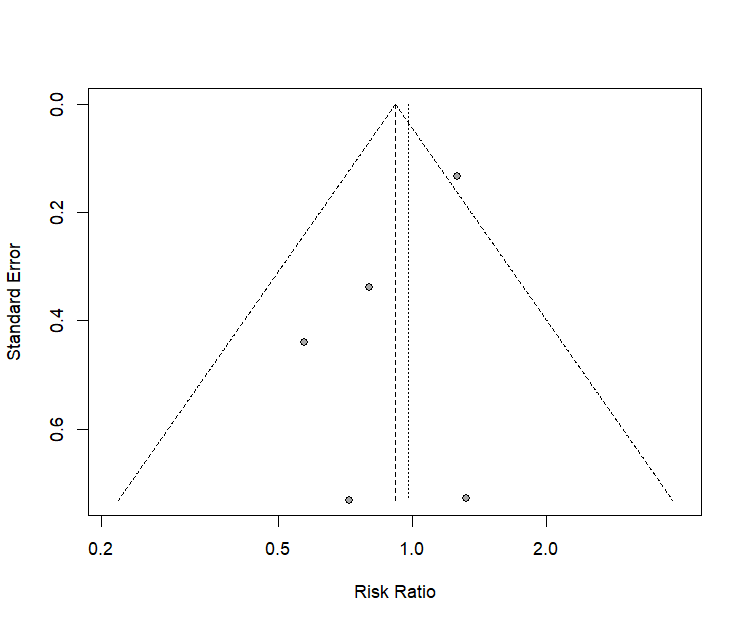

Supplement: Supplementary file 1 — Table S1: MeSH search terms. Figure S1: Funnel plots and heterogeneity estimates of studies evaluating the risk of OSA following radiotherapy. [file HED-48-1587-s001.docx]
